# Adhesion reformation and the limited translational value of experiments with adhesion barriers: A systematic review and meta-analysis of animal models

**DOI:** 10.1038/s41598-019-52457-2

**Published:** 2019-12-03

**Authors:** Chema Strik, Kimberley E. Wever, Martijn W. J. Stommel, Harry van Goor, Richard P. G. ten Broek

**Affiliations:** 1 Department of Surgery, Radboud University Medical Center, Nijmegen, The Netherlands; 2 Systematic Review Centre for Laboratory animal Experimentation (SYRCLE), Department for Health Evidence, Radboud Institute for Health Sciences, Radboud University Medical Center, Nijmegen, The Netherlands

**Keywords:** Reproductive disorders, Motility disorders, Experimental models of disease

## Abstract

Today, 40–66% of elective procedures in general surgery are reoperations. During reoperations, the need for adhesiolysis results in increased operative time and a more complicated convalescence. In pre-clinical evaluation, adhesion barriers are tested for their efficacy in preventing ‘de novo’ adhesion formation, However, it is unknown to which extent barriers are tested for prevention of adhesion reformation. The aim of this systematic review and meta-analysis is to assess the efficacy of commercially available adhesion barriers and laparoscopic adhesiolysis in preventing adhesion reformation in animal models. Pubmed and EMBASE were searched for studies which assessed peritoneal adhesion reformation after a standardized peritoneal injury (in the absence of an intra-peritoneal mesh), and reported the incidence of adhesions, or an adhesion score as outcome. Ninety-three studies were included. No study met the criteria for low risk of bias. None of the commercially available adhesion barriers significantly reduced the incidence of adhesion reformation. Three commercially available adhesion barriers reduced the adhesion score of reformed adhesions, namely Seprafilm (SMD 1.38[95% CI]; p < 0.01), PEG (SMD 2.08[95% CI]; p < 0.01) and Icodextrin (SMD 1.85[95% CI]; p < 0.01). There was no difference between laparoscopic or open adhesiolysis with regard to the incidence of adhesion reformation (RR 1.14[95% CI]; p ≥ 0.05) or the adhesion score (SMD 0.92[95% CI]; p ≥ 0.05). Neither currently commercially available adhesion barriers, nor laparoscopic adhesiolysis without using an adhesion barrier, reduces the incidence of adhesion reformation in animal models. The methodological quality of animal studies is poor.

## Introduction

Postoperative adhesions form after abdominal surgery due to injured peritoneal surfaces and pose a life-long risk of small bowel obstruction, chronic pain, infertility and complications during reoperations^1^. Today, as many as 40–66% of elective procedures in general surgery are reoperations^2–4^. During reoperations, adhesions from previous operations are present in about 90% of the patients, and the need for adhesiolysis results in an increased operative time and a more complicated convalescence. The incidence of iatrogenic bowel injury during adhesiolysis is estimated to be 6–10% and is associated with increased postoperative mortality and healthcare costs^1,4^. Adhesion reformation is of particular clinical importance because the risk of iatrogenic bowel injury and the severity of the adhesions increases exponentially after multiple abdominal operations^5,6^. Adhesions also cause a significant socio-economic burden. Determining the overall costs of adhesions and adhesion-related complications is complex. Only the direct hospital costs for some adhesion-related complications are known. Costs associated with an episode of small bowel obstruction are €16 305 (SD €2 513) for operative treatment, and for non-operative treatment € 2 277 (SD € 265)^7^. Total direct hospital costs in the US have been estimated at 1.3 billion US dollars in 1994 rising to 2.3 billion in 2011^8,9^. A recent cost-effectiveness analysis showed that adhesion barriers are cost-effective in open colorectal, and might be cost-effective in laparoscopy if societal costs are taken into account^10^.”

Peritoneal trauma leads to a denuded surface with submesothelial damage evoking an inflammatory response and a mismatch in the fibrinolytic balance in favor of the persistence of fibrin clots. Under the influence of various cytokines, these fibrin bands are transformed into granulation tissue and subsequently converted into permanent, highly organized, collagenous tissue^11,12^. Evidence suggests that the local expression of growth factors and proteins inhibiting fibrinolysis are increased in the peritoneum of patients with existing adhesions, potentially leading to aggravated adhesion reformation^13,14^. As a result, adhesion barriers may be less efficacious in preventing adhesion reformation. This is supported by a clinical study in which icodextrin 4% did significantly reduced formation of de novo adhesions but not adhesion reformation^15^. A previous systematic review and meta-analysis of clinical studies demonstrated a reduction of clinically relevant consequences of adhesions by hyaluronate carboxymethylcellulose and oxidised regenerated cellulose. However, the included studies predominantly studied the prevention of *de novo* adhesion formation, or did not differentiate between patients with and without existing adhesions at the index operation^16^. Awareness of reduced efficacy of adhesion barriers in preventing adhesion reformation compared to *de novo* adhesion formation is low among surgeons^17^.

During pre-clinical evaluation, the efficacy of adhesion barriers in preventing de novo adhesion formation is tested. However, it is unknown to which extent barriers are tested with regards to their ability to prevent of adhesion reformation. In addition, it is unclear which experimental animal models are most valid for translation to clinical efficacy. In this systematic review and meta-analysis we assess the efficacy of five commercially available adhesion barriers and laparoscopic adhesiolysis in preventing adhesion reformation in animal models. Additionally, the impact of different study characteristics on the incidence of adhesion reformation and severity of adhesions was investigated to provide guidance on the design and execution of future animal studies.

## Methods

### Search strategy, inclusion and exclusion criteria

The review methodology was documented *a priori* in a protocol and was registered on www.syrcle.nl (protocol accessible through website)^18^. On the 11^th^ of June 2017, electronic searches were performed in the Pubmed and Embase databases. The search strategies involved the following search components: “adhesion” and “peritoneum”, “peritoneal adhesion”, “animal”, “prevention”, “adhesiolysis”, “anti-adhesive”, and both the generic and trade names of known adhesion barriers. We combined this with search filters designed specifically to enhance the retrieval of animal studies^19,20^. The complete search strategy can be found in digital Supplement Table S1.

References were screened for eligibility on the basis of title and abstract, by two independent reviewers (CS and MS) in the web-based program EROS^©^ (IECS, Buenos Aires, Argentina). Subsequently, the full-text manuscripts of eligible studies were reviewed for inclusion. In case of doubt in the title and abstract screening phase, the full manuscript was always evaluated. In the full-text screening phase, discrepancies were discussed, and if necessary resolved with the help of a third revierwer (RtB). A study was included if it assessed peritoneal adhesion reformation after a standardized peritoneal injury in animals, in absence of an intra-peritoneal mesh, and reported outcome data on either the incidence of adhesions or an adhesion score. A study was excluded if (1) it was not performed in animals, (2) it was not an original full research article presenting original data, (3) no numerical or graphical data was reported on any of the outcome measures, (4) an intra-peritoneal mesh was used, (5) no adhesion reformation model was used (6) the adhesion barrier was not applied intra-peritoneally, or (7) if the experimental groups were not treated according to the same protocol. If necessary, articles in languages other than English were translated. Studies were excluded from the meta-analysis if there was no control group or no treatment group. In case of missing outcome data or unreported key study details, authors were contacted via e-mail (two attempts). In case the authors did not respond, the study was excluded from the meta-analysis.

### Study characteristics and data extraction

Data extraction was performed by two independent researchers. Discrepancies were discussed, and if necessary resolved with the help of a third reviewer. The following data were extracted from studies included in the systematic review: animal species (rat, rabbit, mouse, other), sex (male, female, mixed, unknown), number of animals in treatment and control groups, number of animals excluded or deceased, reason for exclusion, cause of death, type of model (cecal abrasion, uterine horn, other(for example ischemic button)), type of control group (internal or external), intervention used as control (no intervention, saline, placebo or a combination of these), perioperative treatment with analgesia, antibiotics or fluids for resuscitation, standardized peritoneal injury, timing of intervention (both 1^st^ and 2^nd^, or 2^nd^ operation only), repeated injury at 2^nd^ surgery, and the method of adhesiolysis (sharp and blunt, coagulation). Cecal abrasion as type of model included four variations: cecal abrasion alone, cecal abrasion + sidewall damage, cecal abrasion + ileal damage, and sidewall damage alone. We included the latter because the cecum is often attached to the peritoneal sidewall area after damage. Bibliographic details such as author and year of publication were also registered.

Two outcome measures were assessed: incidence of adhesions and adhesion score. Type of scoring system (tenacity, extent, morphology, mixed) and the minimal and maximal value of the scoring system used were collected.

### Quality assessment

We devised an 8-point scoring system to assess the methodological quality of included articles based on SYRCLE’s risk of bias tool^21^. Our scoring system included (1) presence of an ethical statement, (2) adequate allocation sequence generation, (3) similar groups at baseline, (4) blinded treatment allocation, (5) method of serosal injury specified and standardized, (6) random outcome assessment, (7) blinded outcome assessment and (8) incomplete outcome data adequately addressed. Studies meeting 7 or 8 criteria of this scoring system were considered to have a low risk of bias.

### Data synthesis and statistical analyses

Data were analyzed using STATA 11.2 (StataCorp LLC, Texas, USA), using the ‘metan’ package. Meta-analysis was performed for two outcome measures, the incidence of adhesions and adhesion score. The incidence of adhesions in the control groups was pooled using inverse variance. Additionally, to assess the efficacy of adhesion barriers, the difference between treated and control groups was computed by means of respectively the risk ratio for the incidence of adhesions, and the standardized mean difference (SMD) using Hedges’ *g* for the adhesion score. A random effects model was used to account for expected heterogeneity between the studies. Subgroup analyses were performed on both outcome measures and meta-regression was used to assess significant differences between the subgroups. A minimum of 5 studies was required in each subgroup to perform an analysis. Subgroup analyses were performed for: animal species, sex, type of model, method of adhesiolysis, time interval between surgery, type of scoring system and repeated injury at 2^nd^ laparotomy. The efficacy of commercial available barriers (oxidized regenerated cellulose, hyaluronate carboxymethylcellulose, icodextrin, polyethylene glycol and dextran) was assessed irrespective of the number of studies available for analysis, but without pooling the results across different barriers. Additionally, the efficacy of saline for reducing the incidence of adhesions and the adhesion score was assessed.

When a study contained multiple control groups, the control group undergoing the same surgical procedure without an additional intervention was used. Additional positive control groups were analyzed as treatment groups. In case a research papers described several experimental designs, the different experiments were analyzed as independently. If a study contained multiple dosages or volumes of the same adhesion barrier, the most efficacious dosage or volume was chosen. If multiple treatment groups were compared to the same control group, the number of animals in the control group was divided by the number of treatment groups. When data were presented only graphically, we extracted numerical data from the graphs using ImageJ^©^ digital image analysis software. In case the incidence in the control and treatment group was 100%, the incidence in both groups was reduced with 10% to enable meta-analysis. If the standard error was reported it was converted to the standard deviation for meta-analysis. If the standard deviation was 0, the lowest standard deviation of another group within that study was used in the meta-analysis.

Publication bias was assessed for the overall efficacy of adhesion barriers for the incidence of adhesions and adhesion score using Duval and Tweedie’s trim and fill analysis, and Eggers’ regression analysis for small study effects.

Ethics approval was not necessary due to the nature of this being a systematic review and meta-analysis. All authors approved the publication of this manuscript.

## Results

### Search and study selection

**Figure 1:**
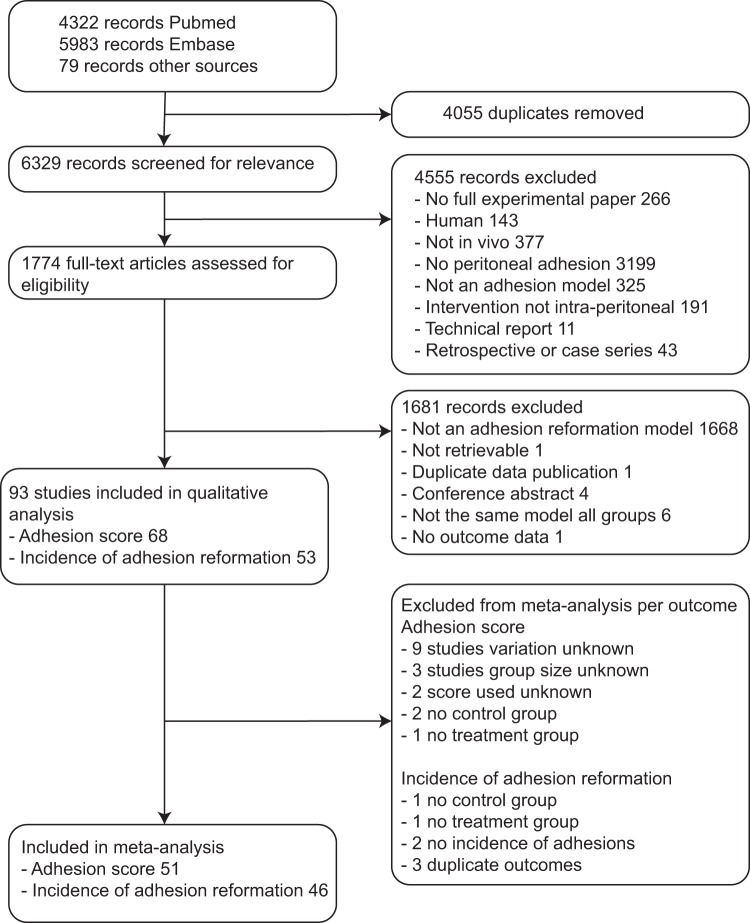
Flow-chart.

The search in PubMed and EMBASE yielded 6329 unique records, of which 4555 were excluded after title and abstract screening. Out of 1774 studies in which approximately 2100 different barriers were tested, 93 (5%) met our inclusion criteria in the full-text screening phase. These studies assessed 48 (2.3%) different barriers for preventing adhesion reformation. All other studies were excluded since they assessed *de novo* adhesion formation, either in the presence of absence an intra-peritoneal mesh (Fig. 1).

### Characteristics of included studies

**Figure 2:**
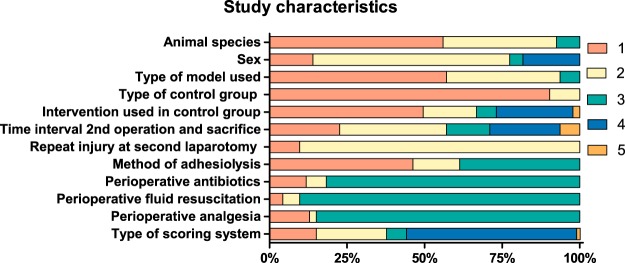
General characteristics of the studies included in the systematic review. *Animal species:* 1 rabbit, 2 rat, 3 other, 4 not applicable (NA), 5 NA; *Sex*: 1 male, 2 female, 3 mixed, 4 unknown, 5 NA; *Type of experimental model*: 1 cecal abrasion, 2 uterine horn, 3 other, 4 NA, 5 NA; *Type of control group*: 1 external control, 2 internal control, 3 NA, 4 NA, 5 NA; *Intervention used in control group*: 1 no intervention, 2 saline, 3 placebo, 4 multiple control groups, 5 other adhesion barrier; *Time interval between 2nd operation and sacrifice*: 1 7 days, 2 14 days, 3 21 days, 4 other, 5 unknown; *Repeated peritoneal injury at second laparotomy*; 1 Yes, 2 No, 3 Unknown, 4 NA, 5 NA; *Method of adhesiolysis*: 1 blunt/sharp, 2 coagulation, 3 unknown, 4 NA, 5 NA; *Perioperative antibiotics*, *fluid resuscitation or analgesia*: 1 Yes, 2 No, 3 Unknown, 4 NA, 5 NA; *Type of scoring system*: 1 tenacity, 2 extent, 3 morphology, 4 combined, 5 other.

The characteristics of the 93 included studies are shown in Fig. 2. Eighty-four studies assessed the efficacy of an adhesion barrier, whereas 9 studies assessed a surgical technique. Fifty-three (57%) studies used a cecal abrasion model, 34 (36%) a uterine horn model and 6 (7%) studies used other experimental models. Out of the 53 cecal abrasion models, 14 used a cecal abrasion only model, 8 a cecal abrasion plus ileal damage, 25 a cecal abrasion plus sidewall injury and 6 studies used a sidewall injury only. Twelve (13%) studies used male animals, 58 (63%) studies used female animals, four (4%) studies used both males and females and in seventeen (19%) studies the sex of the animals was not specified. Eleven (12%) studies applied an adhesion barrier at both surgeries. No study combined laparoscopic adhesiolysis with an adhesion barrier. Fifty-three (57%) studies assessed the incidence of adhesions as outcome measure, of which 46 could be included in meta-analysis. Sixty-eight (73%) studies assessed an adhesion score as outcome measure, of which 51 (55%) could be included in meta-analysis.

### Risk of bias

**Figure 3:**
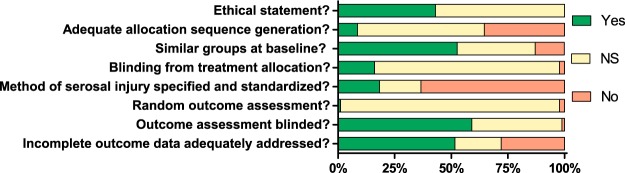
Risk of bias for the studies included in the systematic review. showing the proportion of studies scoring with low risk of bias (yes), high risk of bias (no) or that did not specify (NS) the key methodological variables.

The result of the risk of bias assessment is shown in Fig. 3. No study met the criteria for low risk of bias. Therefore we could not perform a subgroup analysis based on the level of risk of bias. Eight (9%) studies reported and used an adequate randomization method, 52 (56%) studies mentioned randomization but did not specify the method of randomization and 33 (35%) studies did not use an adequate randomization method. Fifty-five (59%) studies adequately blinded the outcome assessment, whereas 1 (1%) study did not specify blinding of the outcome assessment and 37 (40%) studies did not adequately blind the outcome assessment. Fifteen (16%) studies adequately blinded the treatment allocation, in 76 (82%) studies this was not specified and in 2 (2%) studies this was not performed.

### Incidence of adhesions in control groups, efficacy of adhesion barriers and impact of study characteristics on outcome

**Figure 4:**
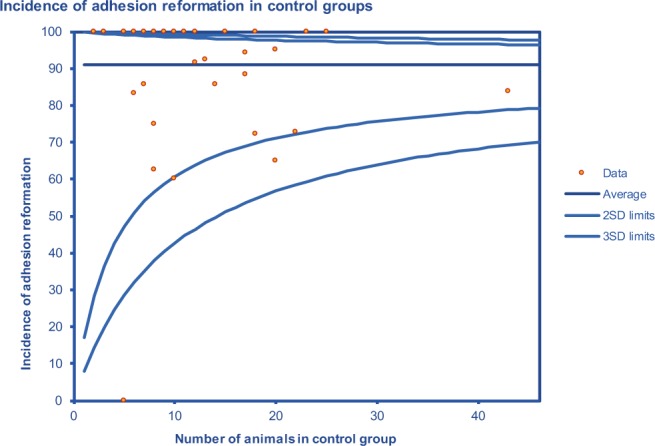
Funnel plot showing the incidence of adhesion reformation as a percentage of the total number of animals in the control group.

The pooled incidence of adhesions in the control groups was 91% (Fig. 4). Only one model did not develop any adhesions at all; this study (Luciano 1989 B) used a laparoscopic peritoneal sidewall abrasion model. The range of incidences of adhesions in control groups, excluding the study performed by Luciano 1989 B, was 60% to 100%.

When pooling all studies (including both commercially available and experimental barriers), adhesion barriers reduced the incidence of adhesion reformation (Risk Ratio 1.35 [95% CI 1.21, 1.50]; p < 0.01) and the adhesion score of reformed adhesions (SMD 1.94 [95% CI 1.61, 2.27]; p < 0.01). The forest plots are shown in the Supplemental File Figures S1, S2. The between-study heterogeneity (I^2^) for the incidence of adhesions and adhesion score was 0% and 70%, respectively.

**Figure 5:**
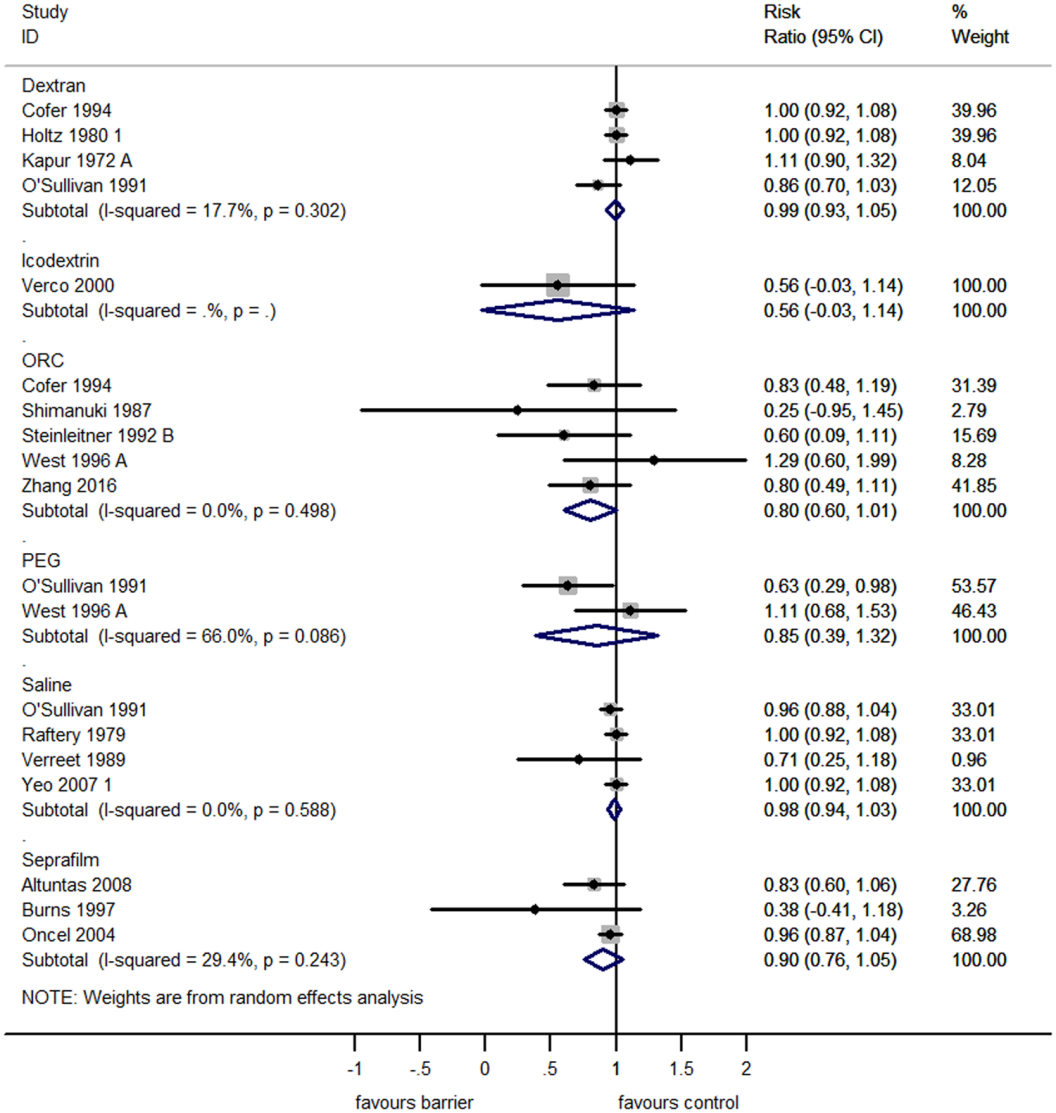
Forest plot showing the efficacy of commercially available adhesion barriers in reducing the incidence of adhesion reformation.

**Figure 6:**
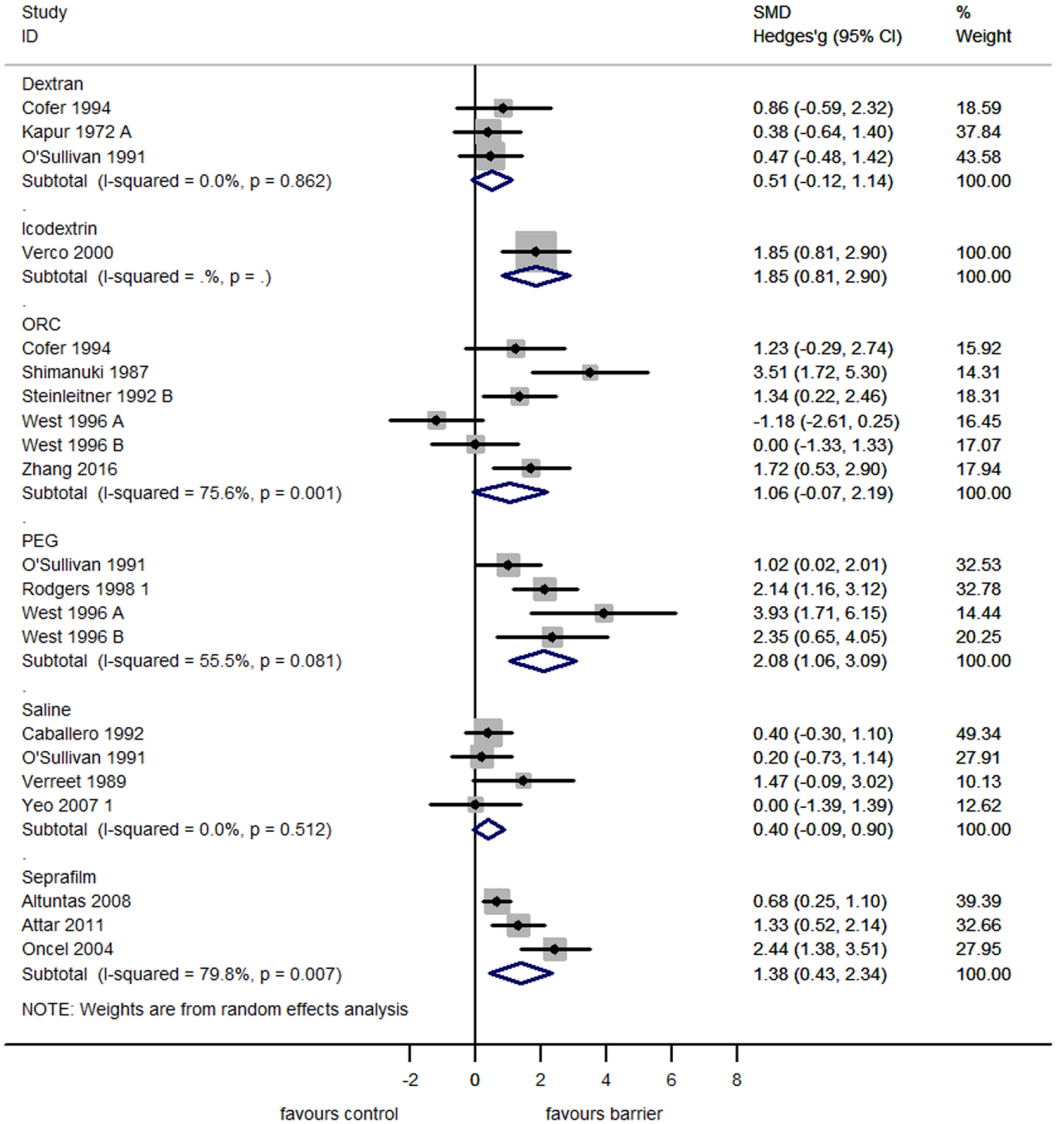
Forest plot showing the efficacy of commercially available adhesion barriers in reducing the adhesion score of reformed adhesions.

When analyzed separately, none of the commercially available adhesion barriers reduced the incidence of adhesion reformation (Fig. 5). Three studies assessed the efficacy of Seprafilm (RR 0.90 [95% CI 0.76, 1.05]), five studies assessed Oxidized Regenerated Cellulose (ORC; RR 0.80 [95% CI 0.60, 1.01]), two studies assessed PEG (RR 0.85 [95% CI 0.39, 1.32]), four studies assessed Dextran (RR 0.99 [95% CI 0.93, 1.05) and one study assessed the efficacy of Icodextrin (RR 0.56 [95% CI −0.03, 1.14]). In contrast to reduction of incidence, three commercially available adhesion barriers individually reduced the adhesion score of reformed adhesions (Fig. 6). Three studies assessed the efficacy of Seprafilm (SMD 1.38 [95% CI 0.43, 2.34]), four studies assessed the efficacy of PEG (SMD 2.08 [95% CI 1.06, 3.09]), one study assessed the efficacy of Icodextrin (SMD 1.85 [95% CI 0.81, 2.90]; p < 0.01). Three studies assessed the efficacy of Dextran (SMD 0.51 [95% CI −0.12, 1.14]), and six studies assessed the efficacy of ORC (SMD 1.06 [95% CI −0.07, 2.19]), and did not significantly reduce the adhesion score of reformed adhesions.

Subgroup analysis of the impact of experimental factors on the incidence of adhesion reformation showed a borderline significant difference in efficacy between studies using rats (RR 0.88 [95% CI 0.80, 0.97]) and rabbits (RR 0.67 [95% CI 0.55, 0.80]). Type of model, sex and repeated peritoneal injury did not explain significant proportions of heterogeneity between studies (Supplement Table S2). Subgroup analysis of the impact of experimental factors on the adhesion score of reformed adhesions showed a significant difference in efficacy between studies using male (SMD 1.15 [95% CI 0.40, 1.91]) and female animals (SMD 2.05 [95% CI 1.68, 2.42]). Type of model, species, repeated peritoneal injury, the time-interval between laparotomies, method of adhesiolysis and the type of adhesion scoring system did not explain significant proportions of heterogeneity between studies (Supplement Table S3). A subgroup analysis on using an adhesion barriers at the first and second surgery versus only at the second surgery could not be performed because of insufficient data

### Publication bias of studies assessing the efficacy of adhesion barriers

Analysis of the funnel plots was performed for the overall efficacy of adhesion barriers, for both outcomes (incidence of adhesions and adhesion score). Egger’s regression analysis showed no significant evidence for publication bias for either outcome measure (incidence of adhesions p = 0.18; adhesion score: p = 0.50). This was in line with the result of the trim and fill analysis, which did not indicate any funnel plot asymmetry or missing data points for either outcome.

### Efficacy of surgical technique

Seven out of nine studies that assessed surgical technique compared the efficacy of laparoscopic versus open adhesiolysis. Three studies used the incidence of adhesion reformation and four studies used an adhesion score as outcome measure. Two studies assessed the efficacy of CO_2_ or Nd:YAG laser versus electro microsurgery (data not pooled).

**Figure 7:**
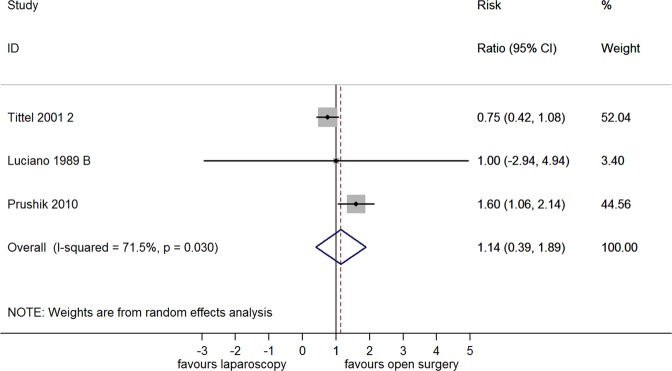
Forest plot showing the efficacy of laparoscopic versus open adhesiolysis in reducing the incidence of adhesion reformation.

There was no difference between laparoscopic or open adhesiolysis with regard to the incidence of adhesion reformation (RR 1.14 [95% CI 0.39, 1.89]; Fig. 7) or adhesion score (SMD 0.92 [−0.04, 1.87]; Supplement Fig S3). The between-study heterogeneity (I^2^) for the incidence of adhesions and the adhesion score was 71.5% and 63.4%, respectively. Publication bias was not assessed due to the limited number of studies available for analysis.

## Discussion

### Concise statement of principal findings

The results of this systematic review show that only 5% of all animal studies on adhesion prevention assessed the efficacy of adhesion barriers or surgical technique for the prevention of adhesion reformation. Furthermore, only 2% of all barriers that were evaluated for their efficacy in preventing adhesion formation were assessed in preventing adhesion reformation. Studies were highly heterogeneous with regard to the employed study characteristics, and overall methodological quality was poor. Our meta-analysis indicates that none of the commercially available adhesion barriers reduces the incidence of adhesion reformation. However, they do reduce the severity of reformed adhesions in animal studies. There is no evidence regarding the efficacy of laparoscopic adhesiolysis in reducing the incidence of adhesion reformation.

### Strengths and limitations of the present study

We were able to include a large number of studies per outcome measure, which enabled us to investigate the effect of several experimental factors. Of note, our review only includes evidence obtained from academic journals. Additional data may be available at the manufacturing companies of the various barriers. If such data would be made available without restrictions and with full transparency they could make a valuable addition to the data presented here. The studies included in our systematic review and meta-analysis were highly heterogeneous. Although the between-study heterogeneity for studies assessing the incidence of adhesion reformation seemed low (I^2^ 0%), this is an artificially low number due to the wide confidence intervals of studies in which the incidence was 100% in the treatment and control group where data was corrected to enable meta-analysis. We have accounted for this heterogeneity by using a random effects model, and investigated potential sources of heterogeneity by performing subgroup analysis. However, differences between subgroups should be interpreted with caution because some subgroups represent only few studies and a limited number of animals. We did not include a correction for multiple hypothesis testing because our aim to comprehensively assess the available pre-clinical evidence and to provide guidance for future (pre)clinical studies rather than using our results for direct clinical implementation. The pooled efficacy analysis, combining experimental and commercial available adhesive barriers, shows they reduce the incidence of adhesion reformation. However, due to the differences between adhesive barriers and the small effect size (OR 0.83) this finding cannot be readily translated into a clinical implication. The Egger’s regression and trim and fill analysis did not observe any significant publication bias. An explanation that at least one adhesion barrier was effective in each study could be the differences in methodological quality, heterogeneity or only publication of positive results^22^. Importantly, this could lead to an overestimation of the effect sizes of the efficacy of adhesion barriers. Despite the potential overestimation, none of the commercially available adhesion barriers reduced the incidence of adhesion reformation in individual analysis.

### Comparison to other studies and clinical implications

The overall methodological quality was low, since no studies met the criteria for low-risk of bias and reporting was poor. Similar results have been found for many animal studies in different medical fields^23–25^. The method of randomization and blinding of treatment allocation was not specified in 56% and 82% of the studies included in this systematic review. This underlines the suboptimal reporting of important study characteristics central to good scientific practice. One study demonstrated that the demands from nine different journals, selected on the basis that they publish a high number of animal studies, for the description of animal studies are limited in a way that studies cannot adequately repeated^26^. It is of great importance that the methodology and reporting of animal studies improve in order to increase the reproducibility and ultimately the translational value of animal studies to clinical practice.

The commercially available adhesion barriers did not significantly reduce the incidence of adhesion reformation. Ninety-three out of 1774 studies assessed the efficacy of adhesion barriers or surgical technique on adhesion reformation and only eleven studies assessed commercially available barriers. Icodextrin was the most poorly studied with only one study assessing its efficacy. Considering the rate of 40 to 66% of reoperations in elective abdominal surgery and the large clinical burden of adhesiolysis, the number of animal studies assessing adhesion reformation is low^2–4^. In a national survey regarding the awareness of adhesions, almost 80% of the surgeons that have used adhesion barriers did so during reoperations for adhesion-related complications. Seprafilm® and Adept® (Icodextrin) were the most used adhesion barriers^17^. The indications for using Seprafilm® as described by the manufacturer do not differentiate between preventing ‘de novo’ adhesions or reformed adhesions whereas the indication for using Adept® is limited to preventing adhesion reformation in gynecological laparoscopic adhesiolysis and is contra-indicated in patients requiring a laparotomy incision, bowel resection, appendectomy or that have peritonitis^27,28^. The efficacy of Adept in reducing the incidence of adhesion reformation after laparoscopic adhesiolysis in gynecologic surgery is questionable and there is no evidence for the effectiveness of Adept in general surgery, which findings compare with our animal data^16,29,30^. Prevention of adhesion reformation is key in reducing the clinical burden of adhesions and both experimental animal and clinical evidence is lacking in how to optimally prevent adhesion reformation by adhesive barriers.

Laparoscopic adhesiolysis did not significantly reduce the incidence of adhesion reformation in the included animal studies. In 3 studies assessing the incidence of adhesion reformation there was a minimal difference between open and laparoscopic adhesiolysis (RR 1.06). It has been reported that the incidence of *de novo* adhesion formation against the incision is reduced after laparoscopic surgery in comparison to open surgery and that laparoscopic surgery is associated with a reduced incidence of adhesive small bowel obstruction^1,31^. A similar efficacy might not apply to adhesion reformation after adhesiolysis. Laparoscopic adhesiolysis as intended operative procedure is limited to improving fertility of women with peri-adnexal adhesions and treating chronic abdominal pain related to abdominal adhesions^32,33^. Our animal data indicate that laparoscopic adhesiolysis alone may not be effective in reducing the incidence of adhesion reformation, and could benefit from usage of an adhesion barrier as adjuvant to improve efficacy in reducing adhesion reformation.

We have found that the type of animal and sex used significantly impact adhesion reformation. We advocate that future studies should use mixed sex populations. When interpreting the results of animal studies assessing the efficacy of adhesion barriers, the large effect sizes in rabbits compared to rats should be taken into account. Possibly, heterogeneity and predominant use of the uterine horn model in rabbits explain the difference. Unfortunately, due to small subgroup size we were not able to further analyze the impact of covariates regarding animal species. No difference in efficacy between studies incorporating a single versus repeated peritoneal injury was observed when assessing the incidence of adhesion reformation. However, we nevertheless propose using a repeated peritoneal injury model, because most reoperations in humans involve peritoneal dissection and subsequent damage^34^. The high heterogeneity of the included studies is reflected in the wide range of 60–100% incidence of adhesion reformation in control groups. It has been recognized that animal studies assessing the efficacy of adhesion barriers are highly heterogeneous and efforts have been made to improve the standardization of animal models by comparing several models of adhesion formation^35^. However, this has not led to any experimental model being consistently employed by several study groups. Only 53 out of 93 studies incorporated the incidence of adhesion reformation as an endpoint of the study. This is a relatively low number considering the clinical importance of this outcome: only the absence of adhesions will preclude complications secondary to adhesiolysis, adhesive bowel obstruction and female infertility. To increase translational value, the primary endpoint of animal studies assessing the efficacy of adhesion barriers should be the overall incidence of adhesions. We have found a pooled incidence of 91% with a range of 60–100% in controls, which can be used for power calculations of future studies. Nearly all studies employed a different method to score adhesions. The method of scoring adhesions showed a tendency towards a larger effect size when only the extent of adhesion was assessed, in comparison to scores based on the tenacity of adhesions, or a combination of tenacity and extent. Attempts to standardize the method of scoring adhesions have been made, however this standardized approach has not yet been universally adopted^36^. Adhesion scores should only be incorporated as secondary endpoints and if scored, the tenacity of adhesions is particularly important, since more tenacious adhesions correlate with an increased risk of iatrogenic organ injury during adhesiolysis^4,37^. Systematic reviews and meta-analyses of animal studies have shown to be a useful tool in improving the design of future animal studies, as well as an aid in the translation of animal studies to clinical trials^38–40^. Our study provides several recommendations for improving experimental studies. Future animal studies testing new anti-adhesive agents should include both *de novo* adhesion prevention and adhesion reformation, use the most challenging model (e.g. the repeated peritoneal injury model), and use adhesion incidence as primary outcome. Furthermore, when designing a clinical study of adhesion prevention with barriers, patients with baseline adhesions should be analyzed separately in order to increase the clarity of results.

## Conclusion

Current commercially available adhesion barriers and laparoscopic adhesiolysis without using an adhesion barrier do not reduce the incidence of adhesion reformation in animal models. Overall, the methodological quality of animal models is poor and there is heterogeneity with regard to the animal models used for assessing the efficacy of adhesion barriers. The results of this systematic review and meta-analysis can be used to aid in the improvement of the design of animal and clinical studies assessing adhesion-related outcomes.

## Supplementary information

Dataset 1

## Data Availability

The datasets used and/or analysed during the current study are available from the corresponding author on reasonable request.
