# Supplementary material for: Adhesion reformation and the limited translational value of experiments with adhesion barriers: A systematic review and meta-analysis of animal models
Source: Sci Rep. 2019 Dec 3;9:18254. doi: 10.1038/s41598-019-52457-2 (PMC6890766; doi:10.1038/s41598-019-52457-2)

Supplementary appendix

Adhesion reformation and the limited translational value of experiments with adhesion barriers: A systematic review and meta-analysis of animal models

Chema Strik, M.D., Kimberley E. Wever PhD, Martijn W.J. Stommel, M.D., PhD, Harry van Goor, M.D., PhD, FRCS, Richard P.G. ten Broek, M.D., PhD

Table of contents

Table S1. Full Search Strategy

Table S2. Subgroup analysis using meta-regression for assessing the impact of experimental factors on the incidence of adhesions,**.**

Table S3. Subgroup analysis for the adhesion score

Figure S1. Pooled analysis of the efficacy of the different adhesion barriers in preventing adhesion reformation

Figure S2. Pooled analysis of the efficacy of adhesion barriers in reducing the score of adhesion reformation

Figure S3. Forest plot showing the efficacy of laparoscopic versus open adhesiolysis in reducing the adhesion score of reformed adhesions

Table S1. Full Search Strategy

Disease of interest:
Adhesions:

(("Tissue Adhesions"[Mesh] OR “Tissue adhesions” [Tiab] OR “Tissue Adhesion” [tiab] OR “Surgical Adhesions” [tiab] OR “Surgical adhesion” [tiab])

COMBINE WITH AND
Peritoneum:

("Peritoneum"[Mesh] OR “peritoneum” [tiab] OR "Mesentery"[Mesh] OR “Peritoneum, Visceral” [tiab] OR “Visceral Peritoneum” [tiab] OR “Peritoneum, Parietal” [tiab] OR “Parietal Peritoneum” [tiab] OR “Cavity, Peritoneal” [tiab] OR "Abdomen"[Mesh] OR “abdomen” [tiab] OR “abdomens” [tiab] OR "Abdominal Cavity"[Mesh] OR “Abdominal Cavities” [tiab] OR “Cavities, Abdominal” [tiab] OR “Cavity, Abdominal” [tiab] OR “Cavitas abdominis” [tiab] OR “intra-abdominal” [tiab] OR “intraabdominal” [tiab] OR “intraperitoneally” [tiab] OR “intraperitoneally” [tiab]))

COMBINE WITH OR
Combined terms for peritoneal adhesions:

(“peritoneal adhesion” [tiab] OR “peritoneal adhesions” [tiab] OR “abdominal adhesion” [tiab] OR “abdominal adhesions” [tiab] OR “intra-abdominal adhesion” [tiab] OR “intra-abdominal adhesions” [tiab] OR “intraabdominal adhesion” [tiab] OR “intraabdominal adhesions” [tiab])

COMBINE WITH AND
Intervention:
"Seprafilm" [tiab] ”Sepracoat” [tiab] OR "INTERCEED" [tiab] OR “RepelCV” [tiab] OR “Gore-tex surgical membrane” [tiab] OR “Gore tex surgical membrane” [tiab] OR "Polytetrafluoroethylene"[Mesh] OR “GORE-TEX” [tiab] OR “GORE TEX” [tiab] OR “Goretex” [tiab] OR “Prevadh” [tiab] OR "SuperSeal" [tiab] OR “Oxidized regenerated cellulose” [tiab] OR “cellulose” [tiab] OR “tc7” [tiab] OR “cellulose” [tiab] OR “Hyaluronate carboxymethylcellulose” [tiab] OR “carboxymethylcellulose” [tiab] OR “hyaluronan” [tiab] OR “hyaluron” [tiab] OR “hyaluronic acid” [tiab] OR "Adcon-P" [tiab] OR “Adept” [tiab] OR “Icodial” [tiab] OR “Baxter Brand of Icodextrin” [tiab] OR “Extraneal” [tiab] OR "icodextrin" [tiab] OR “Sepracoat” [tiab] OR "Seprafilm" [tiab] OR “Tisseel” [tiab] OR "Fibrin Tissue Adhesive" [Mesh] OR “Fibrin Adhesive” [tiab] OR “Fibrin Glue” [tiab] OR “Fibrinogen Adhesive” [tiab] OR “Fibrin Sealant System” [tiab] OR “Crosseal” [tiab] OR “Fibrin Klebe System Immuno” [tiab] OR “Transglutine” [tiab] OR “Fibrin Sealant” [tiab] OR “Tissel” [tiab] OR “Tissucol” [tiab] OR “Beriplast” [tiab] OR “Fibrin Seal” [tiab] OR “Sprayshield” [tiab] OR “Spraygel” [tiab] OR “PEG” [tiab] OR “polyethylene glycol” [tiab] OR “Intercoat” [tiab] OR "intergel" [tiab] OR “Sepraspray” [tiab] OR "crystalloid solutions" [tiab] OR “Ringer’s lactate” [tiab] OR "Isotonic Solutions"[Mesh] OR "Sodium Chloride"[Mesh] OR “Sodium Chloride” [tiab] OR “NaCl” [tiab] OR “Saline Solution” [tiab] OR “adhesiolysis” [tiab]

COMBINE WITH AND
Domain Animals:
Animal search filter as published by SYRCLE*
***** Hooijmans, C.R., et al., *Enhancing search efficiency by means of a search filter for finding all studies on animal experimentation in PubMed.* Lab Anim, 2010. **44**(3): p. 170-5.

Table S2. Subgroup analysis using meta-regression for assessing the impact of experimental factors on the incidence of adhesions

Subgroup	Number of studies	Effect size	95% confidence interval		Heterogeneity	
			lower	upper	I^2 residual	p
**Experimental model**						
Cecal abrasion‡	31	0.80	0.73	0.87		
Uterine horn	7	0.90	0.69	1.12		
Other*	3					
Between subgroup					0.00	0.41
**Animal species**						
Rabbit	19	0.67	0.55	0.80		
Rat	16	0.88	0.80	0.97		
Other*‡	6					
Between subgroup					0.00	0.05
**Sex**						
Female‡	17	0.79	0.66	0.92		
Male	8	0.85	0.75	0.94		
Mixed*	3					
NS	13					
Between subgroup					0.00	0.21
**Repeated peritoneal injury**						
No‡	36	0.84	0.78	0.91		
Yes	5	0.62	0.37	0.86		
Between subgroups					0.00	0.09

*NS not specified, ‡ reference category, *not performed due to the low number of studies with this study characteristic*

Table S3. Subgroup analysis for the adhesion score

Subgroup	Number of studies	Effect size	95% confidence interval		Heterogeneity	
			lower	upper	I^2 residual	p
**Experimental model**						
Cecal abrasion	25	1.66	1.24	2.08		
Uterine horn‡	19	2.01	1.51	2.51		
Other*	2					
Between subgroup					70.43	0.45
**Animal species**						
Rabbit	25	1.87	1.44	2.31		
Rat	15	1.99	1.42	2.56		
Other*‡	6					
Between subgroup					68.09	0.56
**Sex**						
Female‡	29	2.05	1.68	2.42		
Male	6	1.15	0.40	1.91		
Mixed*	3					
NS*	8					
Between subgroup					63.46	0.03
**Repeated peritoneal injury**						
No‡	38	1.81	1.47	2.16		
Yes	8	1.91	1.10	2.71		
Between subgroups					70.48	0.84
**Time between surgery**						
7 days	12	2.21	1.58	2.83		
14 days	17	1.53	1.03	2.03		
21 days	6	2.02	1.20	2.84		
Other*‡	11					
Between subgroups					67.16	0.30
**Method adhesiolysis**						
Blunt and sharp	20	1.77	1.28	2.27		
Coagulation	7	1.87	1.02	2.72		
NS*	19					
Between subgroups					70.22	0.96
**Type of adhesion scoring system**						
Tenacity‡	10	1.40	0.76	2.05		
Extent	16	2.15	1.63	2.67		
Combination	18	1.63	1.16	2.10		
Other*	2					
Between subgroups					65.04	0.09

*NS not specified, ‡ reference category, *not performed due to the low number of studies with this study characteristic or because subgroup is not specified*

Figure S1. Pooled analysis of the efficacy of the different adhesion barriers in preventing adhesion reformation

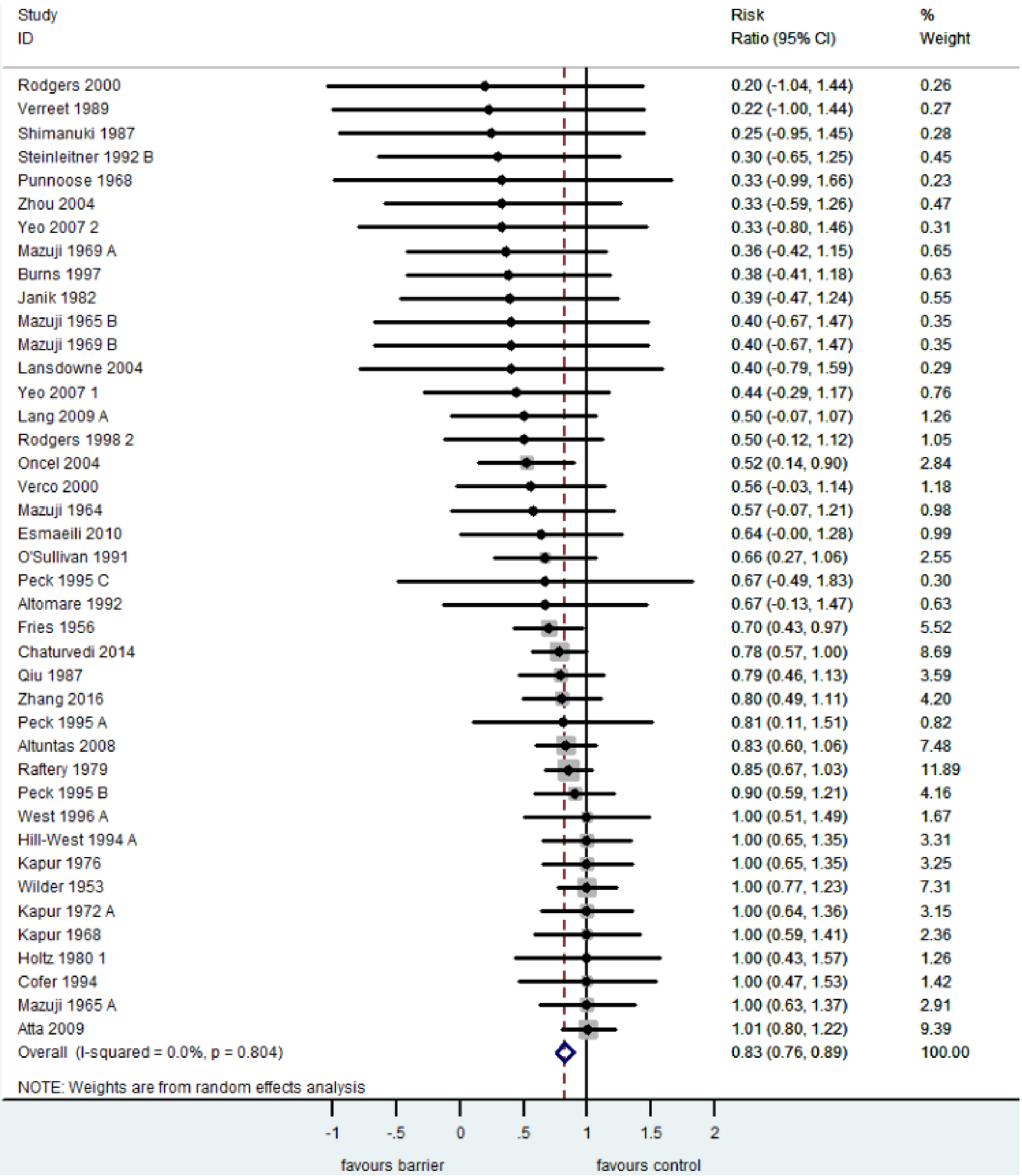

Figure S 2 Pooled analysis of the efficacy of adhesion barriers in reducing the score of adhesion reformation

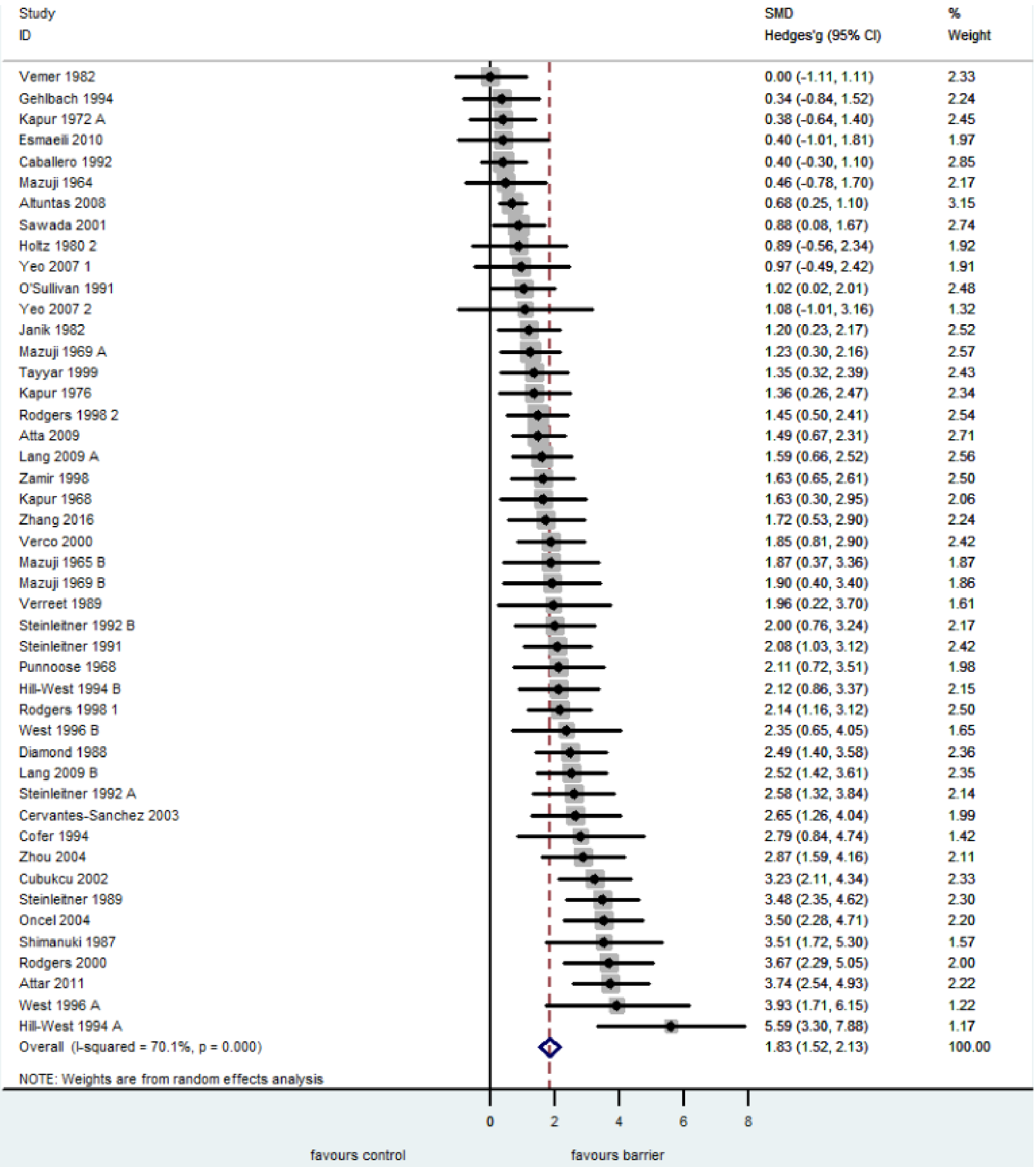

Figure S3. Forest plot showing the efficacy of laparoscopic versus open adhesiolysis in reducing the adhesion score of reformed adhesions

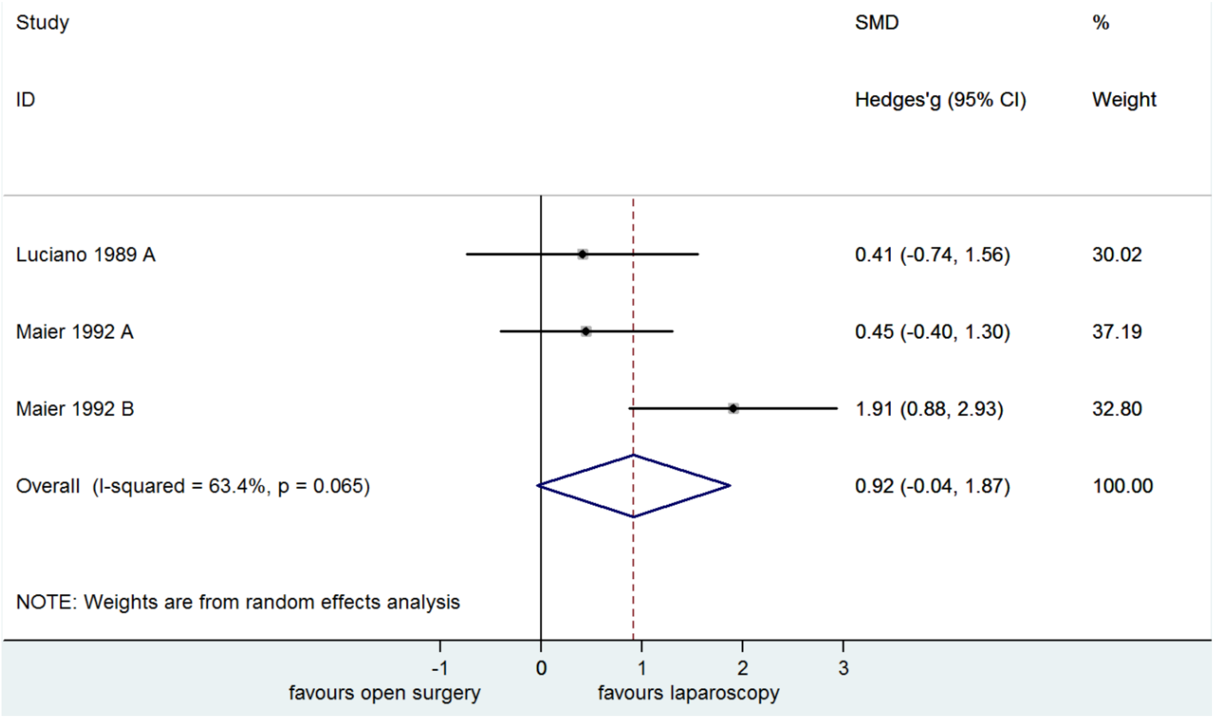

Supplement: Supplementary file 1 — Dataset 1 [file 41598_2019_52457_MOESM1_ESM.pdf]
